# Greater decision-making competence is associated with greater expected-value sensitivity, but not overall risk taking: an examination of concurrent validity

**DOI:** 10.3389/fpsyg.2015.00717

**Published:** 2015-05-28

**Authors:** Andrew M. Parker, Joshua A. Weller

**Affiliations:** ^1^RAND CorporationPittsburgh, PA, USA; ^2^School of Psychological Science, Oregon State UniversityCorvallis, OR, USA; ^3^Decision ResearchEugene, OR, USA

**Keywords:** decision making, competence, expected value sensitivity, individual differences, risky choice

## Abstract

Decision-making competence reflects individual differences in the susceptibility to committing decision-making errors, measured using tasks common from behavioral decision research (e.g., framing effects, under/overconfidence, following decision rules). Prior research demonstrates that those with higher decision-making competence report lower incidence of health-risking and antisocial behaviors, but there has been less focus on intermediate processes that may impact real-world decisions, and, in particular, those implicated by normative models. Here we test the associations between measures of youth decision-making competence (Y-DMC) and one such process, the degree to which individuals make choices consistent with maximizing expected value (EV). Using a task involving hypothetical gambles, we find that greater EV sensitivity is associated with greater Y-DMC. Higher Y-DMC scores are associated with (a) choosing risky options when EV favors those options and (b) avoiding risky options when EV favors a certain option. This relationship is stronger for gambles that involved potential losses. The results suggest that Y-DMC captures decision processes consistent with standard normative evaluations of risky decisions.

## Introduction

Behavioral decision research illuminates how individuals’ actual decisions systematically deviate, on average, from how they *should* behave according to normative theories (Hastie and Dawes, 2010). However, considerable heterogeneity across individuals is apparent for even the most robust effects, such as susceptibility to framing effects, sunk costs, and under/overconfidence (e.g., Klayman et al., 1999; Stanovich, 1999; Levin et al., 2002). Researchers have begun to consider the degree to which stable individual differences in susceptibility to these biases exist, and if these differences predict psychosocial outcomes. An emerging literature on *decision-making competence* suggests not only that a latent variable representing this over-arching cognitive competence exists, but also that it demonstrates predictive validity across a range of problematic real-world behaviors, including health-risking sexual behavior, delinquency, and self-reported decision outcomes (Parker and Fischhoff, 2005; Bruine de Bruin et al., 2007).

Although these results shed light on the association between decision-making competence and risk behavior, it remains unclear how decision-making competence relates to component evaluation processes that may lead to making a risky choice. According to psychological risk-return models, risk behavior is based on both perceptions of uncertainty (i.e., risk perceptions) and expected benefits, of the choice options (i.e., evaluation of expected benefits; Weber and Johnson, 2008). Given that the latter also theoretically corresponds with decision making based on a normative standard, we predict that choosing options that have a more favorable expected value (EV), when considering risky choices, will be associated with higher decision-making competence. In a large sample of 18–19 year old community residents, we repurpose a once-considered, but later abandoned, decision-making task to examine the degree to which decision-making competence is associated with EV sensitivity for risky decisions involving either potential gains or losses. If this hypothesis is supported, it would reinforce DMC’s conceptual foundation of capturing deviations from normative decision making.

### Shifting From Path Independence to Risky Choice and EV Sensitivity

Parker and Fischhoff’s (2005) original study found positive correlations across several well-documented decision-making tasks (resisting framing effects, resisting sunk costs, appropriate confidence in knowledge, consistent risk perceptions, applying decision rules, and recognizing social norms), suggesting an underlying latent construct labeled “Youth Decision-Making Competence” (Y-DMC), as well as correlations between Y-DMC and a comprehensive set of behavioral covariates and outcomes, including executive cognitive functioning. However, Parker and Fischhoff (2005) also included another measure, path independence, which was not consistently associated with neither the other Y-DMC indices, nor the behavioral covariates and outcomes. Subsequent similar null findings eventually led to the removal of this measure from subsequent assessments (Bruine de Bruin et al., 2007).

For the path independence task, respondents’ choices between certain outcomes and risky gambles (e.g., winning $50 for sure versus a 50% chance to win $100, or $0) were judged for their consistency with the rational-choice axiom of path independence^1^, but no other normative criteria. Because the path independence task presents respondents with choices between simple gambles and certain gains or losses of varying that differed in the relative EV between choice options, measures of EV sensitivity and risky choice (i.e., preference) can be derived.

### EV Sensitivity for Potential Gains and Losses

Research has suggested relevant group-level differences in the ability to make EV-sensitive decisions. For example, using an expanded version of the “Cups” task, a repeated-measures decision-making task involving EV-sensitive risky decisions for potential gains and losses, patients with focal lesions to the ventromedial prefrontal cortex were especially EV-insensitive (Weller et al., 2007). Research also suggests considerable developmental differences in EV-sensitivity, in which young children make less EV-sensitive choices than older children, whereas older adults demonstrate lower EV sensitivity compared to younger adults (e.g., Schlottmann and Tring, 2005; Henninger et al., 2010; Weller et al., 2011). Moreover, Weller and Fisher (2012) found that maltreated children (10–11 years) were less EV sensitive than a same-age community comparison group, particularly to avoid certain losses (c.f., Weller et al., 2015b).

A broader literature suggests potential asymmetries may arise when considering risky decisions involving achieving potential gains versus avoiding potential losses. Prospect theory, for instance, states that individuals tend to be more risk-averse for gains and risk-seeking for losses (Kahneman and Tversky, 1979). Developmental studies have suggested that the ability to make EV-sensitive judgments when losses are involved may develop later than for potential gains (Schlottmann and Tring, 2005; Levin et al., 2007; Weller et al., 2011). Moreover, neuropsychological research suggests that losses produce greater autonomic arousal and attention-orienting responses compared to equal gains (Satterthwaite et al., 2007; Rubaltelli et al., 2012), greater frontal-cortical activation (Gehring and Willoughby, 2002), and may activate a more complex neural processing system (Kuhnen and Knutson, 2005). Additionally, decisions involving losses have been associated with longer decision response latencies than ones involving potential gains (Xue et al., 2009). Taken together, these results suggest that losses dominate attention (see Yechiam and Hochman, 2013 for a review), and that making EV-sensitive choices in the face of potential losses may require greater deliberative processing capacities in order to disengage from the immediate threat of the potential loss.

### Associations between Y-DMC, EV Sensitivity, and Risk Behavior

Based on this research, we propose that individual differences in cognitive capabilities may be associated more strongly with the more cognitive component of risk evaluation, EV sensitivity, than overall risk perceptions, which are more likely to involve affective, gut-level reactions regarding the behavior (e.g., Weber et al., 2002). Because Y-DMC is conceptualized as reflecting a more reasoned, deliberative approach to decisions, we expect that higher Y-DMC will be associated with greater EV sensitivity, but not necessarily overall risk taking. Supporting this assertion, Weller et al. (2015a) found that decision-making competence was associated more strongly with self-reported perceptions of expected benefits than with perceptions of risk across several risk domains. However, in that study, expected benefits were assessed through subjective assessments rather than through behavioral assessment of EV sensitivity. Further, choosing in accordance with EV has been associated with indices of intelligence and executive cognitive function in a manner similar to decision-making competence, suggesting similar underlying processes (e.g., Henninger et al., 2010; Donati et al., 2014; Webb et al., 2014). Finally, because of the asymmetries in processing gains and losses described above, we predict that the correlation between EV-sensitivity and Y-DMC will be stronger for potential losses, relative to potential gains.

## Methods

### Participants

These data were collected through the Center for Education and Drug Abuse Research (CEDAR) as part of a larger longitudinal study. Participants were recruited at 9–13 years of age and were assessed approximately every 2–3 years. The current data are from the age 18–20 assessment (Wave 4). Further description of the CEDAR design can be found in Tarter and Vanyukov (2001).

Five hundred twenty-five CEDAR respondents completed all Y-DMC component measures^2^. Respondents were mostly male (65.1%), on average 18.9 years old (SD = 0.5), 76.5% had a high school diploma/GED, and 73.7% self-identified as Caucasian^3^.

### Procedure and Measures

At the age 18–19 CEDAR assessment, participants completed the Y-DMC measure, including the repurposed path independence task.

#### Youth Decision-Making Competence

Youth decision-making competence consists of six tasks, selected to cover skills central to normative decision-making theories (Parker and Fischhoff, 2005). *Resistance to Framing* measures whether choices are affected by formally irrelevant variations in how options are described (e.g., condom effectiveness described in terms of success or failure rates) and includes five pairs of items (two pairs include risky choices). Performance equals the number of consistent choice pairs. *Resistance to Sunk Cost* uses two single-choice problems to assess the willingness to abandon an irrecoverable prior investment (i.e., sunk cost), considering only future consequences. Performance equals the number of such choices. *Consistency in Risk Perception* assesses the extent to which sets of risk judgments are internally consistent (e.g., the judged chance of dying from any cause “in the next year” should be no larger than the same risk judged “between now and when you turn 30”). Performance is the number of internally consistent item sets (out of 5). *Applying Decision Rules* assesses the accurate application of specific decision rules to seven hypothetical choices among products characterized on multiple attributes within a table format. Performance equals the number of correctly applied decision rules. *Under/overconfidence* uses a 42-item true/false knowledge questionnaire, in which each answer is accompanied by a probabilistic confidence judgment (50% = just guessing; 100% = absolutely sure). Scores equal the absolute value of the difference between participant’s mean confidence judgment and the percent of correct true/false responses. *Recognizing Social Norms* reflects a calibration between a respondent’s estimation of the degree to which an undesirable behavior is normative (e.g., “out of 100 people your age, how many would say it is sometimes ‘OK’ to steal under certain circumstances”) and the actual percent of respondents who endorsed that “it is sometimes ‘OK’ to engage in each behavior.” Scores represent each person’s within-subject correlation between judged norms and observed norms. Finally, a composite Y-DMC score is calculated by the mean of standardized component scores.

#### Risky-Choice Task

Participants were asked to make hypothetical choices (i.e., no real payoffs) for 18 simple gambles between a certain option and a risky option (e.g., win $50 for sure, 50% win $100, otherwise win $0; see Supplementary Material). For each, an indifference option (“doesn’t matter to me”) was also provided. Choices varied in whether they involved either gains or losses, outcome magnitude (potentially ranging from zero to $100), and the EV of gamble relative to the certain option. Within gain and loss domains, some decisions involved choices in which the certain option is greater than the EV of the gamble (i.e., risk disadvantageous), for some the EVs were equated (i.e., equal EV), and for some the gamble had greater EV (i.e., risk advantageous). There were nine trials for each domain, with three trials for each EV category. The range of relative EV between choice options were equated, in terms of absolute magnitude, for gains and losses.

Expected value sensitivity is the sum of choices in which the participant selected the more favorable EV option for risk-advantageous and risk-disadvantageous trials. Respondent choices are scored a 1 if the chosen option has the more favorable EV and zero otherwise. We also create summary variables for the number of risky choices made on risk-advantageous, equal EV, and risk-disadvantageous trials, for gains and losses, respectively. A risky choice is coded +1 if the respondent chose the risky option, 0 if the respondent chose “doesn’t matter to me,” and -1 if the respondent chose the certain option^4^. Each measure represents a sum of risk-taking for three decisions, and ranges between -3 and +3. Thus, positive scores represent an overall preference for risky options, whereas negative scores represent a preference for certainty.

## Results

Descriptive statistics for Y-DMC are supplied in Supplementary Material.

### Expected-Value Sensitivity

The upper portion of **Table 1** shows descriptive statistics for the EV-sensitivity indices. Overall, respondents chose the more favorable EV option 56.3% of the time. We found that individuals displayed greater EV sensitivity for the gain domain than the loss domain, *t*(522) = 5.35, *p* < 0.001, *d* = 0.23^5^.

**Table 1:** Expected value (EV) Sensitivity and its correlation with youth decision-making competence (Y-DMC).

	EV sensitivity		
Summary	Gain domain	Loss domain	Overall
**Descriptive statistics**
Potential range	0–6	0–6	0–12
Mean (SD)	3.55 (1.29)	3.21 (1.60)	6.76 (2.50)
**Correlation with Y-DMC measure**
Resistance to Framing	0.03	0.13^a∗∗^	0.10^∗^
Resistance to Sunk Cost	-0.06	0.06^a^	0.01
Consistency in Risk Perception	0.05	0.11^a∗∗^	0.10^∗^
Applying Decision Rules	0.16^∗∗∗^	0.29^a∗∗∗^	0.27^∗∗∗^
|Under/overconfidence|	0.10^∗^	0.18^a∗∗∗^	0.17^∗∗∗^
Recognizing Social Norms	0.19^∗∗^	0.20^∗∗^	0.22^∗∗∗^
Y-DMC Composite	0.15^∗∗∗^	0.31^a∗∗∗^	0.28^∗∗∗^

^***^p < 0.001; ^**^p < 0.01; ^*^p < 0.05; ^+^*p* < 0.10. ^a^Represents a significant difference between gain and loss trials, at *p* < 0.05, one-tailed, following Steiger’s (1980) procedure.

The lower portion of **Table 1** shows the associations between EV Sensitivity and the Y-DMC tasks and composite score. Consistent with our predictions, we found that Y-DMC scores were associated with EV Sensitivity. Although we find a positive association between Y-DMC composite scores and overall EV Sensitivity, the effect size for the association between the Y-DMC composite and EV Sensitivity for the loss domain is double in magnitude compared to that observed for the gain domain. Next, we followed Steiger’s (1980) *z*-score procedure to test for differences between dependent correlations. The correlation between composite Y-DMC scores and EV Sensitivity was significantly greater for the loss domain than the gain domain. This pattern is robust across the component measures, except for Recognizing Social Norms, in which the correlations were nearly identical across domains. Resistance to Sunk Costs was not associated with EV Sensitivity in either domain, but the change from a non-significant negative to a non-significant positive correlation was significant.

### Risky Choice

The upper portion of **Table 2** provides descriptive statistics for the six risky-choice composites. Respondents were more risk-seeking (i.e., positive scores) when risk was advantageous and risk-averse when risk was disadvantageous. Collapsing across the different EV levels, individuals were generally risk averse for the gain domain (*M* = -2.58, SD = 4.62) and risk seeking in the loss domain (*M* = 2.43, SD = 4.69). Furthermore, irrespective of domain, respondents were risk-seeking for risk-advantageous trials (*M* = 1.61, SD = 2.91), were indifferent for equal-EV trials (*M* = -0.10, SD = 2.56), and avoided risks for risk-disadvantageous trials (*M* = -1.66, SD = 2.80). Overall, respondents were relatively risk neutral (summing across all measures in **Table 1**; *M* = -0.15, SD = 6.60).

**Table 2:** Risk choice and its correlation with Y-DMC.

	**Gain domain**			**Loss domain**		
**Y-DMC Measure**	**Risk Advantageous**	**Equal Expected Value**	**Risk Disadvantageous**	**Risk Advantageous**	**Equal Expected Value**	**Risk Disadvantageous**
**Descriptive statistics**						
Potential range	-3–3	-3–3	-3–3	-3–3	-3–3	-3–3
Mean (SD)	0.08 (2.15)	-1.04 (1.71)	-1.62 (1.62)	1.53 (1.67)	0.94 (1.85)	-0.04 (2.09)
**Correlation with Y-DMC measure**						
Resistance to Framing	0.01	0.04	-0.02	0.10^a*^	0.07	-0.04
Resistance to Sunk Cost	-0.04	0.02	-0.02	-0.01	-0.05	-0.09^*^
Consistency in Risk Perception	-0.01	-0.01	-0.03	0.04	0.04	-0.04
Applying Decision Rules	0.03	-0.08^+^	-0.11^*^	0.16^a***^	0.02^a^	-0.13^**^
|Under/overconfidence|	0.02	0.00	-0.11^*^	0.13^a**^	0.04	-0.11^**^
Recognizing Social Norms	0.08^+^	0.02	-0.05	0.16^a***^	0.12^a**^	0.01
Y-DMC Composite	0.03	0.00	-0.11^*^	0.19^a***^	0.08	-0.13^**^

^***^p < 0.001; ^**^p < 0.01; ^*^p < 0.05; ^+^*p* < 0.10. ^a^Represents a significant difference between gain and loss trials, at *p* < 0.05, one-tailed, following Steiger’s (1980) procedure.

The lower portion of **Table 2** presents correlations between risky choice and Y-DMC. For three scales (Resistance to Framing, Resistance to Sunk Cost, and Consistency in Risk Perception), we observed no systematic pattern of correlations with risk-taking. However, Applying Decision Rules, Under/overconfidence, and the Y-DMC composite correlate with greater risk-taking, primarily in the loss domain. However, inspection of the correlations suggest that this correlation is likely due to those demonstrating greater Y-DMC being more likely to take the gamble for risk-advantageous trials than those with lower Y-DMC scores. Thus, this resulted in a null relationship between Y-DMC and overall risk-taking (i.e., total number of risky choices; *r* = 0.01, ns.).

## Discussion

Y-DMC was designed to capture deviations from normative decision making, so demonstrating concurrent validity with another normative decision-making tendency, EV sensitivity, not only widens its “nomological network” of related constructs (Cronbach and Meehl, 1955), but also reinforces the conceptual foundation of the decision-making competence construct. Those displaying greater Y-DMC scores demonstrate greater EV sensitivity, choosing gambles in risk-advantageous situations and avoiding them when risk is disadvantageous. In contrast, Y-DMC scores are not associated with overall risk-taking or on equal EV trials. For equal EV trials in particular, a decision maker should be indifferent between the risky and certain options, and there is no normative solution. With respect to psychological risk-return models (see Weber and Johnson, 2008), these results suggest that decision-making competence may impact risk behaviors via differences in the evaluation of EV, rather than one’s perceptions of risk (Weller et al., 2015a).

Also, consistent with our expectations, Y-DMC scores, and especially the Applying Decision Rules, Under/overconfidence, and Recognizing Social Norms subscales, are more strongly associated to EV sensitivity when considering potential losses. These findings support prior research revealing processing differences for potential gains and losses. Potential losses are believed to direct attention and evoke stronger autonomic arousal (Yechiam and Hochman, 2013). In order to make more EV-sensitive choices for losses, greater deliberative processing resources may be needed to disengage from the emotional impact of realizing a certain loss. Considering research that demonstrates associations between cognitive executive function and both DMC and advantageous decision making (e.g., Parker and Fischhoff, 2005; Del Missier et al., 2012; Webb et al., 2014), we propose that these broader cognitive processes may support normative decision making. As seen elsewhere, however, more deliberative processing does not always lead to more normative or advantageous behavior (e.g., Reyna, 2004), so future research should explore the dynamics among these constructs.

Although promising, the current results should be interpreted in light of several limitations. First, because of the correlational nature of this study, the data cannot speak strongly to the psychological mechanisms that may support normative decision making. Second, these are also secondary data analyses, and therefore limited to existing measures. For example, the path independence task includes a limited set of risky choices, which restricts the range of possible EV sensitivity and risk preferences. Additionally, certain Y-DMC subscales have psychometric limitations. For instance, both the Y-DMC Resistance to Framing and Resistance to Sunk Cost indices demonstrate relatively lower internal consistency compared to other measures, potentially attenuating observed correlations (Parker and Fischhoff, 2005). Both of these limitations have been recognized and addressed in later measures (Bruine de Bruin et al., 2007; Weller et al., 2014). However, given the secondary nature of these data, those improved versions were not available. Additionally, other scales that have been associated with tasks related to DMC, such as numeracy (Weller et al., 2015c), were also not available. Future research using a refined set of measures could further establish the validity of the DMC construct.

Finally, although we conceptualize DMC as a stable construct, it does not imply that normative decision skills cannot be learned or improved. Recent work has demonstrated such malleability. In a direct approach, Jacobson et al. (2012) demonstrated experimentally how decision education can improve decision-making competence. More indirectly, interventions that develop skills that may underlie and support advantageous decision making, such as emotional regulation and goal setting, have shown promise. For example, Weller et al. (2015b) found that adolescent girls in foster care who received such an intervention at approximately 11 years old demonstrated better EV sensitivity approximately 5 years later, whereas girls who received only traditional foster care services over this period were relatively EV-insensitive for both risks involving potential gains and losses.

In closing, the results reinforce our conceptualization and the construct validity of decision-making competence, demonstrating concurrent validity with another metric of normative responding (EV sensitivity). By doing so, these results contribute to a growing literature that aims to identify individual differences in decision making and link these to important psychosocial outcomes.

## Author Contributions

Dr. Parker had primary responsibility for design and data acquisition. Both authors contributed to the analysis, interpretation, and writing for the research described here.

## Conflict of Interest Statement

The authors declare that the research was conducted in the absence of any commercial or financial relationships that could be construed as a potential conflict of interest.

## Footnotes

1 Path independence specifies that choices should be based on outcomes and their likelihood, rather than the sequences of events leading to those outcomes.

2 Eight respondents had incomplete data and were dropped from this analysis.

3 Education information was missing for one respondent.

4 We also explored the potential of scoring responses 1 only if they chose the gamble and 0 otherwise (essentially lumping indifference responses with certain-option responses). Results using this coding were qualitatively similar to those presented below.

5 Following guidance from Dunlop et al. (1996) for calculating effect sizes for correlated designs, we used the independent-samples *t*-test value rather than the paired-samples *t*-test value because the latter is believed to overestimate effect size estimates.
