# Supplementary material for: Greater decision-making competence is associated with greater expected-value sensitivity, but not overall risk taking: an examination of concurrent validity
Source: Front Psychol. 2015 May 28;6:717. doi: 10.3389/fpsyg.2015.00717 (PMC4446538; doi:10.3389/fpsyg.2015.00717)
Supplement: Supplementary file 1 [file Data_Sheet_1.PDF]

Greater decision-making competence reflected in greater expected-value sensitivity, but not overall risk-taking: An examination of concurrent validity

Supplementary Material

The measure of EV Sensitivity was based on the following 18 questions, which were excerpted from a longer scale of 30 questions.

In each of the following problems, choose between flipping a coin and a sure thing. If they both seem the same to you, choose "Doesn't Matter."

1. Which do you like best, (1), (2), or (3)?

(1)	(2)	(3)
Flip a Coin	*Sure Win*	*Doesn't Matter to Me*
If Heads, win **$100**	Win **$50** for sure	
If Tails, win **$0**		
________	________	________

2. Which do you like best, (1), (2), or (3)?

(1)	(2)	(3)
Flip a Coin	*Sure Win*	*Doesn't Matter to Me*
If Heads, win **$100**	Win **$60** for sure	
If Tails, win **$0**		
________	________	________

3. Which do you like best, (1), (2), or (3)?

(1)	(2)	(3)
Flip a Coin	*Sure Win*	*Doesn't Matter to Me*
If Heads, win **$100**	Win **$40** for sure	
If Tails, win **$0**		
________	________	________

4. Which do you like best, (1), (2), or (3)?

(1)	(2)	(3)
Flip a Coin	*Sure Win*	*Doesn't Matter to Me*
If Heads, win **$10**	Win **$5** for sure	

If Tails, win **$0**

________ ________ ________

5. Which do you like best, (1), (2), or (3)?

(1)	(2)	(3)
Flip a Coin	*Sure Win*	*Doesn't Matter to Me*
If Heads, win **$10**	Win **$6** for sure	
If Tails, win **$0**		
________	________	________

6. Which do you like best, (1), (2), or (3)?

(1)	(2)	(3)
Flip a Coin	*Sure Win*	*Doesn't Matter to Me*
If Heads, win **$10**	Win **$4** for sure	
If Tails, win **$0**		
________	________	________

The next questions are about losses.

7. Which do you like best, (1), (2), or (3)?

(1)	(2)	(3)
Flip a Coin	*Sure Loss*	*Doesn't Matter to Me*
If Heads, lose **$100**	Lose **$50** for sure	
If Tails, lose **$0**		
________	________	________

8. Which do you like best, (1), (2), or (3)?

(1)	(2)	(3)
Flip a Coin	*Sure Loss*	*Doesn't Matter to Me*
If Heads, lose **$100**	Lose **$60** for sure	
If Tails, lose **$0**		
________	________	________

9. Which do you like best, (1), (2), or (3)?

(1)	(2)	(3)
Flip a Coin	*Sure Loss*	*Doesn't Matter to Me*
If Heads, lose **$100**	Lose **$40** for sure	
If Tails, lose **$0**		
________	________	________

10. Which do you like best, (1), (2), or (3)?

(1)	(2)	(3)
Flip a Coin	*Sure Loss*	*Doesn't Matter to Me*
If Heads, lose **$10**	Lose **$5** for sure	
If Tails, lose **$0**		
________	________	________

11. Which do you like best, (1), (2), or (3)?

(1)	(2)	(3)
Flip a Coin	*Sure Loss*	*Doesn't Matter to Me*
If Heads, lose **$10**	Lose **$6** for sure	
If Tails, lose **$0**		
________	________	________

12. Which do you like best, (1), (2), or (3)?

(1)	(2)	(3)
Flip a Coin	*Sure Loss*	*Doesn't Matter to Me*
If Heads, lose **$10**	Lose **$4** for sure	
If Tails, lose **$0**		
________	________	________

In each of the next questions, a coin will be flipped to see if you get a choice or not. Without knowing the result of the first flip, what would you choose in each of the following situations?

25. First Flip:

Flip a Coin
If Heads, get the **Choice** below
If Tails, **don't** get the Choice below, win **$0**

Choice: Before the first flip, which do you like best, (1), (2), or (3)?

(1)	(2)	(3)
Flip a Coin	*Sure Win*	*Doesn't Matter to Me*
If Heads, win **$100**	Win **$50** for sure	
If Tails, win **$0**		
_______	_______	_______

26. First Flip:

Flip a Coin
If Heads, get the **Choice** below
If Tails, **don't** get the Choice below, win **$0**

Choice: Before the first flip, which do you like best, (1), (2), or (3)?

(1)	(2)	(3)
Flip a Coin	*Sure Win*	*Doesn't Matter to Me*
If Heads, win **$100**	Win **$60** for sure	
If Tails, win **$0**		
_______	_______	_______

27. First Flip:

Flip a Coin
If Heads, get the **Choice** below
If Tails, **don't** get the Choice below, win **$0**

Choice: Before the first flip, which do you like best, (1), (2), or (3)?

(1)	(2)	(3)
Flip a Coin	*Sure Win*	*Doesn't Matter to Me*
If Heads, win **$100**	Win **$40** for sure	
If Tails, win **$0**		
_______	_______	_______

The next questions are about losses.

28. First Flip:

Flip a Coin
If Heads, get the **Choice** below
If Tails, **don't** get the Choice below, lose **$0**

Choice: Before the first flip, which do you like best, (1), (2), or (3)?

(1)	(2)	(3)
Flip a Coin	*Sure Win*	*Doesn't Matter to Me*
If Heads, lose **$100**	Lose **$50** for sure	
If Tails, lose **$0**		
_______	_______	_______

29. First Flip:

Flip a Coin
If Heads, get the **Choice** below
If Tails, **don't** get the Choice below, lose **$0**

Choice: Before the first flip, which do you like best, (1), (2), or (3)?

(1)	(2)	(3)
Flip a Coin	*Sure Win*	*Doesn't Matter to Me*
If Heads, lose **$100**	Lose **$60** for sure	
If Tails, lose **$0**		
_______	_______	_______

30. First Flip:

Flip a Coin
If Heads, get the **Choice** below
If Tails, **don't** get the Choice below, lose **$0**

Choice: Before the first flip, which do you like best, (1), (2), or (3)?

(1)	(2)	(3)
Flip a Coin	*Sure Win*	*Doesn't Matter to Me*
If Heads, lose **$100**	Lose **$40** for sure	
If Tails, lose **$0**		
_______	_______	_______

Table S1. Descriptive statistics for Y-DMC composite and component tasks.

Measure	Observed Range	Mean	Standard Deviation
Resistance to Framing	0 to 1	.69	.22
Resistance to Sunk Cost	0 to 1	.36	.35
Consistency in Risk Perception	0 to 1	.83	.20
Applying Decision Rules	.29 to 1.00	.89	.17
|Under/overconfidence|	.59 to 1.00	.92	.07
Recognizing Social Norms	-.34 to .89	.52	.22
Y-DMC Composite	-2.27 to 1.21	.00	.52
